# Intrinsic and extrinsic drivers of transmission dynamics of hemorrhagic fever with renal syndrome caused by Seoul hantavirus

**DOI:** 10.1371/journal.pntd.0007757

**Published:** 2019-09-23

**Authors:** Yidan Li, Bernard Cazelles, Guoqing Yang, Marko Laine, Zheng X. Y. Huang, Jun Cai, Hua Tan, Nils Chr. Stenseth, Huaiyu Tian

**Affiliations:** 1 State Key Laboratory of Remote Sensing Science, College of Global Change and Earth System Science, Beijing Normal University, Beijing, China; 2 IBENS, UMR 8197 CNRS-ENS Ecole Normale Supérieure, Paris, France; 3 International Center for Mathematical and Computational Modeling of Complex Systems (UMMISCO), IRD-Sorbonne Université, Bondy, France; 4 Huludao Municipal Center for Disease Control and Prevention, Huludao, Liaoning, China; 5 Finnish Meteorological Institute, Helsinki, Finland; 6 College of Life Sciences, Nanjing Normal University, Nanjing, China; 7 Ministry of Education Key Laboratory for Earth System Modelling, Department of Earth System Science, Tsinghua University, Beijing, China; 8 School of Biomedical Informatics, the University of Texas Health Science Center at Houston, Houston, Texas, United States of America; 9 Centre for Ecological and Evolutionary Synthesis (CEES), Department of Biosciences, University of Oslo, Blindern, Oslo, Norway; 10 Ministry of Education Key Laboratory for Earth System Modeling, Department of Earth System Science, Tsinghua University, Beijing, China; University of Florida, UNITED STATES

## Abstract

Seoul hantavirus (SEOV) has recently raised concern by causing geographic range expansion of hemorrhagic fever with renal syndrome (HFRS). SEOV infections in humans are significantly underestimated worldwide and epidemic dynamics of SEOV-related HFRS are poorly understood because of a lack of field data and empirically validated models. Here, we use mathematical models to examine both intrinsic and extrinsic drivers of disease transmission from animal (the Norway rat) to humans in a SEOV-endemic area in China. We found that rat eradication schemes and vaccination campaigns, but below the local elimination threshold, could diminish the amplitude of the HFRS epidemic but did not modify its seasonality. Models demonstrate population dynamics of the rodent host were insensitive to climate variations in urban settings, while relative humidity had a negative effect on the seasonality in transmission. Our study contributes to a better understanding of the epidemiology of SEOV-related HFRS, demonstrates asynchronies between rodent population dynamics and transmission rate, and identifies potential drivers of the SEOV seasonality.

## Author summary

Seoul hantavirus (SEOV) infections are common in Europe and Asia where a considerably high seroprevalence among the population is found. However, only relatively few hemorrhagic fever with renal syndrome (HFRS) cases are reported. Comprehensive epidemiological data is necessary to study the patterns and drivers of this underestimated disease. Here, we analyzed rodent host surveillance and seroprevalence data from 1998 to 2015 for disease outbreaks in Huludao City, one of the typical SEOV-endemic areas for HFRS in China. Our mathematical models quantified the drivers on HFRS transmission and estimated the epidemiological parameters. Our study provides an understanding of its ecological process between intrinsic and extrinsic factors, human-rodent interface and disease dynamics.

## Introduction

There are 60,000–100,000 cases of hemorrhagic fever with renal syndrome (HFRS), a rodent-borne zoonosis, reported annually, globally [1, 2]. HFRS is caused by subtypes of hantavirus, each of which is associated with a distinct rodent species [3]. Humans are usually infected by inhaling air contaminated with saliva, feces, or urine from infected rodents [4]. China is the main epidemic region for HFRS, and accounts for 90% of cases, globally [1]. The two predominant hantavirus strains circulating in endemic areas of China are Hantaan virus (HTNV), which is carried by striped field mice (*Apodemus agrarius*), and Seoul virus (SEOV), which is carried by Norway rat (*Rattus norvegicus*) [5].

HFRS epidemics vary significantly across seasons and are influenced by extrinsic factors across regions, like climate [6–10]. Following the Hantavirus Pulmonary Syndrome outbreaks that occurred in the Four Corners region of the southwestern United States in 1993, the correlation between hantavirus infections and climatic conditions was described using a cascade hypothesis [11]. The hypothesis posited that favorable climate conditions would lead to more available food, and to greater rodent population sizes, thereby enhancing the risk of hantavirus infections. Similarly, the 2007 HFRS outbreak in temperate southern Europe may have been caused by increased population density of the bank vole, the vector of Puumala virus (PUUV), which, in turn, may have resulted from abundant food due to preceding warmer than usual autumn and winter weather [12, 13]. In central China, higher temperature and precipitation in the previous summer led to favorable food conditions for the striped field mouse, the rodent host of HTNV, which led to an autumn peak in incidence of HFRS [14].

Hantaviruses carried by species of wild rodents, such as HTNV and PUUV, have been extensively studied, but SEOV is vectored by the Norway rat, a domestic rodent that is found in urban environments and is associated with humans [15–17]. SEOV infections, have milder symptoms and lower fatality rates (1–2% vs. 5–10%) than HTNV infections, so less attention is focused on SEOV [16, 17]. Besides, SEOV infections have a higher asymptomatic infection rate of 8–20%, compared with HTNV at 1–4% [18]. In the past two decades, SEOV-related HFRS cases have been reported in the United Kingdom (2012) [19], France (2014) [20], the United States, and Canada (2017) [21] and most provinces of China [22–26]. It has been estimated that more than 25% of hantavirus infections in South Korea and China are caused by SEOV [27, 28]. To better control the potential threat, it is important to understand how critical factors impact SEOV-related HFRS transmission dynamics.

Here, we explore the roles of related intrinsic and extrinsic factors in transmission dynamics of HFRS caused by SEOV in the pre-vaccination era. Intrinsic factors refer mainly to herd immunity or host population dynamics, and extrinsic factors include external forces such as climatic or environmental factors. We collected detailed epidemiological and rodent trapping data from a typical SEOV-endemic area of China, Huludao City, where the Norway rat is the dominant rodent species in residential areas [29]. Additionally, we investigated potential causal relationships among the environmental variables, the population dynamics of the rodent reservoir, and the incidence of HFRS. We subsequently constructed a mathematical model to quantify intrinsic transmission dynamics and extrinsic effects.

## Materials and methods

### Data

Huludao City (40°56′N, 120°28′E) in the Liaoning Province of China (S1 Fig) has a temperate monsoon climate with a hot and rainy summer (mean temperature, 23 °C; mean monthly precipitation, 123 mm) and a cold and rainless winter (mean temperature, -5.7 °C; mean monthly precipitation, 2 mm). The city covers 10,434 square km and has a population of 2.6 million people. HFRS has always posed a severe threat to human health in Huludao City since 1984 [30]. To control HFRS transmission, mass vaccination campaigns against hantavirus were conducted beginning in 2005, and combined with rat extermination programs during 2005 and 2012 [31].

Local demographic data were collected from the Liaoning Statistical Yearbooks. Land cover changes from 1998 to 2015 in Huludao City were derived from the annual European Space Agency (ESA) Climate Change Initiative (CCI) land cover maps, with a 300 m spatial resolution. Meteorological data for Huludao City from 1998 to 2015 were obtained from the Chinese Bureau of Meteorology, and then processed into monthly climate data, including monthly mean maximum temperature, mean temperature, mean minimum temperature, cumulative precipitation, relative humidity, and absolute humidity. Absolute humidity is calculated from temperature and relative humidity [32].

Data of human HFRS cases in Huludao City from 1998 to 2015, were obtained from the Huludao City Center for Disease Control and Prevention, China. All HFRS cases were confirmed according to the standard diagnosis set out by the Ministry of Health of the People’s Republic of China, and by detecting antibodies against hantavirus in serum samples. Serum samples were tested for immunoglobulin (Ig) G and IgM antibodies against HTNV and SEOV by indirect immunofluorescent assay (IFA) [33]. Serum-epidemiological surveys on human hantavirus infection were conducted annually between 1998 and 2015. Anonymous (non-personal) information was used.

Rodent population density was investigated indoors and outdoors by the powder-trace method on a monthly basis, obtained from the historical literature [34]. According to the criteria, 400 powdered panels are placed in the special sites for spot checks across the city every month, of which 240 panels are set in restaurant, hotel, station and other public places where rodents are commonly found, and 160 panels are set in general household. Two powdered panels were placed in each room (about 15 m^2^) in selected spots. Outdoors, each panel was placed along a wall at 5–10 m intervals. All panels were set at night and recovered before sunrise. Rodent population density was calculated as the number of positive powdered panels (with footprints or tail tracks from rodents) divided by the number of effective powdered panels used.

### Ethics statement

The study protocol was reviewed by the institutional review board of the Huludao Municipal CDC and ethics approval was not required. We have received consent from home/land owners to collect rodent data on private land and in private homes. The Animal Ethics Committee of the Huludao Municipal CDC also waived approval for this study.

### Wavelet analysis

Wavelet analysis is widely used in ecology and epidemiology studies to explore the variety in the periodicity of a time series through its decomposition properties [35]. Here, the Morlet wavelet was used to detect the non-stationary characteristics of the incidence of HFRS fluctuations over time, and the bootstrapping method [36] was performed to test the statistical significance of the results. In the significance test, 1000 surrogate datasets were simulated by bootstrapping to test the null hypothesis, where a *P* value of < 0.05 was considered to be statistically significant.

### Convergent cross mapping

Convergent cross mapping (CCM) is used to detect nonlinear causal relationships between time series of HFRS incidence (Y) and environmental factors (X), which is designed to measure causality in nonlinear dynamical systems. CCM can help to identify bidirectional causality (i.e. X and Y are mutually coupled) or unidirectional causality (e.g., X time series variable in a system has a causal influence on Y, but not vice versa) [37, 38]. In this study, CCM was used to identify time-delayed effects of a causal interaction between time series of environmental variability, the population dynamics of rodent hosts, and HFRS incidence based on nonlinear state space reconstruction [39]. In a system where *x* causes *y*, Sugihara *et al*. purposed that the state of *x(t)* can be estimated from the reconstruction of *y(t)* using the nearest-neighbor forecasting method, also called cross mapping [40]. Pearson’s correlation between the estimated *x(t)* and observed *x(t)* reflects the causal effect of *x* on *y*, called “cross map skill” [39]. In analysis, the embedding dimension (E) were set according to the simplex projection results [40]. Time-delayed effect (tp) were set as 0–6 months for detecting the time lags [41].

Given the seasonality of HFRS incidence, climate variables, and rodent population density, the seasonal component and the response of HFRS risk and rodent population density to the anomalies of other variables were examined to avoid spurious correlations. For each variable, 1500 surrogate time series having the same degree of shared seasonality, but with randomized anomalies, were generated for seasonal surrogate test. For a specific variable, the month of year average (seasonal cycle) was calculated first, and the seasonal anomaly was represented by the difference between the observed value and the seasonal cycle. Then the random shuffling of the time series data of seasonal anomalies was added back to the season average [42]. The above analysis was implemented in the “rEDM” package in R.

### The SEOV-related HFRS transmission model

We estimated the epidemiological parameters for HFRS epidemics in Huludao City by fitting the time series from the observed monthly incidence and rodent population density to a discrete-time susceptible-infection model in the Bayesian framework (Table 1) [43].

**Table 1:** The goodness of fit for the candidate models for Norway rat population and HFRS dynamics.

Climate variable	R^2^	DIC
**Norway rat population dynamic model**		
AH_t-3_	0.50	9.32
TMIN_t-4_	0.50	9.17
AH_t-3_, TMIN_t-4_	0.47	11.41
none	0.50	9.17
**HFRS dynamic model**		
RH_t-1_	0.75	7.57
none	0.72	7.89

#### Norway rat population dynamic model

First, we modelled the Norway rat population dynamic model based on Verhulst-Pearl logistic growth model [44], without consideration of SEOV infection, and identified the effect of climate on population fluctuation. Population fluctuations are regulated by an identical seasonal birth rate *(b)*, natural mortality *(m)*, environmental carrying capacity *(K)*, and potential extrinsic effect (*θ*_*r*_) in Eq (1). There is no significant sex difference in the Norway rat population in our study area [45]. To reduce the dimensionality of the model, age classes were omitted.

$$R_{N,t+1}=R_{N,t}+bR_{N,t}-(m\text{+}\theta_{r}+\frac{R_{N,t}}{K})R_{N,t}$$ (1)

Here, 1 month is used as the time interval in the time-discrete model; *b* represents an array consisting of 12 values, representing the reproduction rates of the rodent population over 12 months. As Norway rat breeds throughout the year in the regions studied [45], all of the reproduction rates were set at >0. *m* denotes the natural mortality of the Norway rat population, whose mean life span is generally not more than 1 year [46]. *K* represents the environmental carrying capacity and is used to model the environmental pressure from intraspecific competition for food availability or other resources. For identifying the extrinsic effect on Norway rat population fluctuation, we constructed *θ*_*r*_ to reflect the potential climate effect. Based on the results of the correlation analyses and CCM, minimum temperature and absolute humidity were potential drivers for rodent population dynamics. We constructed equation *θ*_*r*_ = *δ*_*1*_*TMIN*_*t-4*_*+δ*_*2*_*AH*_*t-3*_*+ε*, where TMIN and AH refer to monthly mean minimum temperature and absolute humidity, respectively, and the parameters *δ*_*1*_ and *δ*_*2*_ quantify the degree of climate effect and *ε* is an intercept.

#### Integrated SEOV-related HFRS transmission model

The mathematical model for host rodent population outlined in Eq (1) can be extended to incorporate rodent-human SEOV transmission. SEOV infections were modeled as occurring among rodents and from rodents to humans in Eqs (2)–(4). The rodent host population (*R*_*N*_) was divided into two classes: susceptible (*R*_*S*_) and infected (*R*_*I*_). A susceptible rodent could be infected when bitten by an infectious rodent or by inhaling air contaminated with SEOV in the excrement from infectious rodents [47].

$$R_{S,t+1}=R_{S,t}+bR_{N,t}-(m\text{+}\theta_{r}+\frac{R_{N,t}}{K})R_{S,t}-R_{S,t}\beta_{R}R_{I,t}$$ (2)

$$R_{I,t+1}=R_{I,t}\text{+}R_{S,t}\beta_{R}R_{I,t}-(m+\theta_{r}+\frac{R_{N,t}}{K})R_{I,t}$$ (3)

$$H_{I,t+1}=\rho H_{S,t}\beta_{t}\frac{{\left(R_{I,t}+\tau \right)}^{\alpha }}{H_{N,t}}$$ (4)

In the above equations, all newborn rats were regarded as being susceptible to infection because there is no vertical transmission of SEOV [47]. Given that there is evidence that SEOV infection has no evident impact on death and fertility in Norway rat [48], we did not differentiate mortality (*m*) and birthrate *(b)* between *R*_*S*_ and *R*_*I*._ *β*_*R*_ is the transmission rate between rodents and *α* allows for non-linear contacts from rodents to humans. *H*_*N*_ represents the whole human population and *H*_*S*_ represents susceptible human population. Observed cases *Y*_*I*_ are assumed to be correlated to the unobserved number of cases according to a binomial observation process with constant observation rate *ρ*, due to non-reporting from mild symptoms overlooked by the health system and asymptomatic infections. The prior information of *ρ* (0.006, 0.002–0.009) was assessed from our epidemiological survey. *β*_*t*_ represents SEOV transmission, which contains seasonal contact rates between rodents and humans *β*_*sea*_ and climate-driven transmission potential *β*_*cli*_, *β*_*t*_ = *β*_*sea*_*β*_*cli*_. Based on the results of the correlation analyses and CCM, a causal relationship between relative humidity and HFRS incidence was detected. Therefore, we constructed that *β*_*cli*_ = *δ*_*3*_*RH γ t-1*, where *RH*_*t-1*_ was relative humidity with 1-month lag, and *δ*_*3*_ was used to quantify the effect of relative humidity on HFRS transmission dynamics and *γ* allowed for the non-linearity of the effect. The results of the estimation for *γ and δ*_*3*_ were the mainly focused where the direction of the climate effect depends on whether *γ (δ*_*3*_*>0)* is positive or negative. All parameter settings are shown in Table 2.

**Table 2:** The prior setting and posterior probability for parameters in the optimal model.

Description	Par.	Prior/Range	Posterior mean	Std
**Norway rat population dynamic model**				
Environment carrying ability	*K*	12 [5, 25]	8.24	1.11
Natural mortality	*m*	0.07 [0.04, 0.5]	0.16	0.08
Initial rodent population	*R*_*N*,*0*_	1.4 [0.5, 3]	1.31	0.24
Reproduction rate in Jan.	*b(1)*	1 [0.01, 5]	0.83	0.16
Feb.	*b(2)*	1 [0.01, 5]	0.90	0.16
Mar.	*b(3)*	1 [0.01, 5]	1.06	0.17
Apr.	*b(4)*	1.5 [0.01, 5]	0.93	0.16
May.	*b(5)*	1.5 [0.01, 5]	0.77	0.15
Jun.	*b(6)*	1 [0.01, 5]	1.24	0.19
Jul.	*b(7)*	1 [0.01, 5]	1.14	0.17
Aug.	*b(8)*	1 [0.01, 5]	0.85	0.17
Sep.	*b(9)*	1.2 [0.01, 5]	0.93	0.17
Oct.	*b(10)*	1.2 [0.01, 5]	1.22	0.18
Nov.	*b(11)*	1 [0.01, 5]	1.15	0.18
Dec.	*b(12)*	1 [0.01, 5]	0.98	0.18
**HFRS dynamic model**				
Scale factor	*δ*_*3*_	0 [0, +∞]	17.68	4.58
Forcing factor	*γ*	0 [-∞, +∞]	-1.23	0.20
Initial virus-carrying rodent	*R*_*I*,*0*_	0.1 [0, 1.5]	0.02	0.01
Infectious rate (*R*_*I*_, *R*_*S*_)	*β*_*R*_	1 [0.01, 1]	0.14	0.02
Error term of rodent population	*τ*	0 [0, 3]	2.24	0.21
Observation rate	*ρ*	0.006 [0.002, 0.009]	0.006	0.002
Nonlinear effect of rodent population	*α*	1[0, 100]	9.07	1.15
Seasonal contact rate (*R*_*I*_, human) in Jan.	*β*_*sea (1)*_	1 [0.01, 100]	47.12	3.45
Feb.	*β*_*sea (2)*_	1 [0.01, 100]	89.26	3.86
Mar.	*β*_*sea (3)*_	5 [0.01, 100]	84.24	3.77
Apr.	*β*_*sea (4)*_	5 [0.01, 100]	87.09	3.72
May.	*β*_*sea (5)*_	5 [0.01, 100]	54.16	3.16
Jun.	*β*_*sea (6)*_	1 [0.01, 100]	65.05	3.42
Jul.	*β*_*sea (7)*_	1 [0.01, 100]	41.96	5.71
Aug.	*β*_*sea (8)*_	1 [0.01, 100]	32.56	5.13
Sep.	*β*_*sea (9)*_	3 [0.01, 100]	40.22	5.25
Oct.	*β*_*sea (10)*_	3 [0.01, 100]	40.75	4.41
Nov.	*β*_*sea (11)*_	3 [0.01, 100]	42.73	4.33
Dec.	*β*_*sea (12)*_	5 [0.01, 100]	45.98	4.46

Std, standard deviation of the sample mean.

The Markov chain Monte Carlo (MCMC) sampling method was used for parameter estimation with the MATLAB toolbox delayed rejection adaptive metropolis algorithm [49]. The values of the parameters were estimated by sampling from the convergent posterior distributions of the Markov chains. Gelman-Rubin-Brooks MCMC convergence diagnostic was used to test convergence by identifying whether the series came from a stable distribution or not [50]. Prior distributions of the parameters were mainly set according to scientific literature and values of biological relevance, but those parameters that lacked prior information were set to a large variance (100) with no boundaries (Table 2). The chains were set to perform 1 million iterations with burn-in of 20,000. Deviance information criterion (DIC) was used to measure the goodness of fit for the models with various framework, and a difference value >10 was regarded to be significant. R^2^ was used to measure the interpretability of the model for the observed data.

## Results

### HFRS epidemics in Huludao City

**Fig 1:**
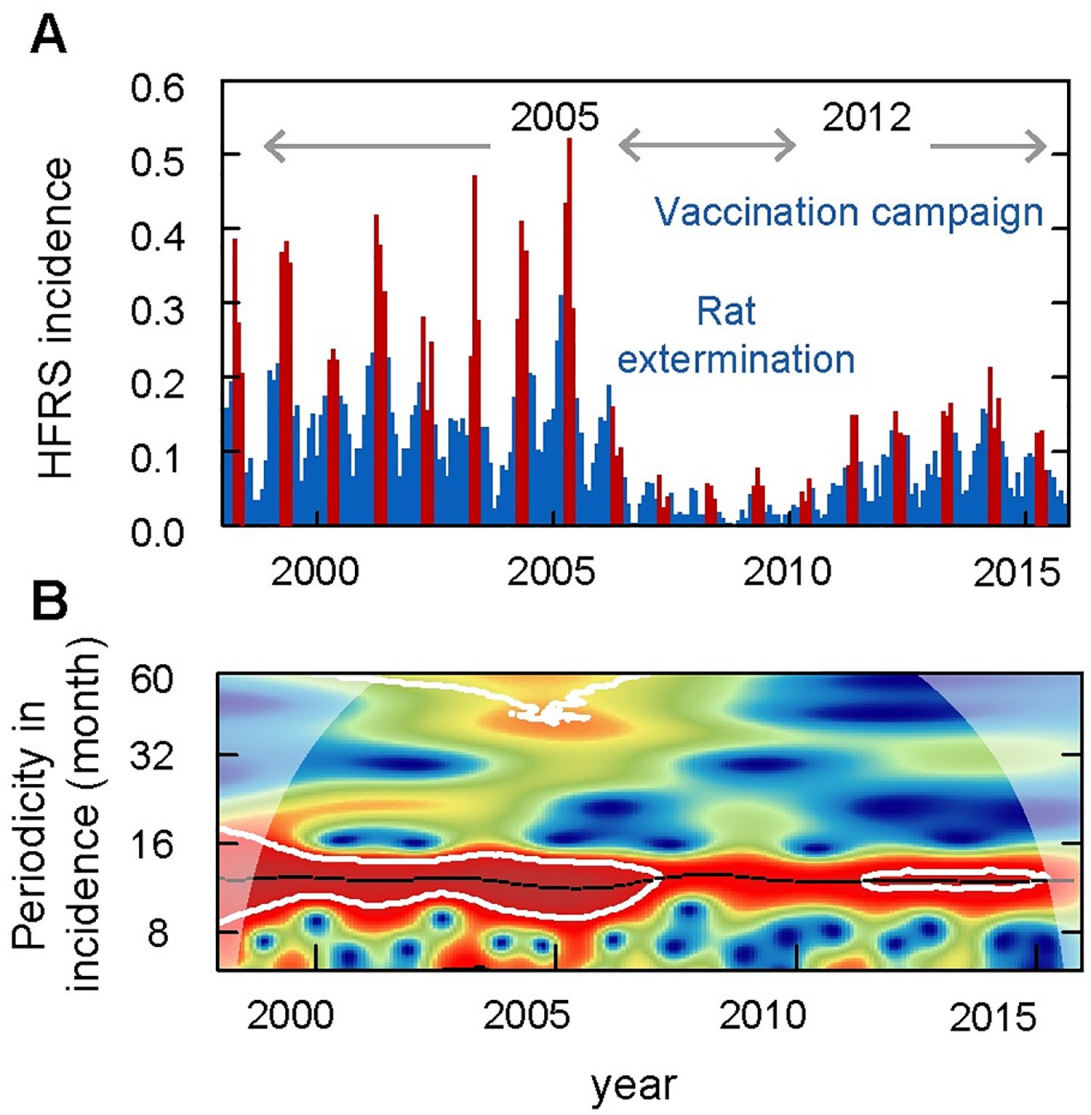
Seasonality and periodicity of hemorrhagic fever with renal syndrome (HFRS) incidence in Huludao City, 1998 to 2015. (A) HFRS incidence per month. Red bars represent disease incidence in spring (March to May), while blue bars represent the other months. (B) Periodic variety of HFRS incidence. Colors indicate the power of the wavelet, where red to blue represent strong to weak power and the black line indicates the maximum power. The white line represents statistical significance (P < 0.05).

During 1998 to 2015, 6796 HFRS cases were reported in Huludao City, with an annual incidence from 2.50 (1/100,000, minimum in 2008) to 26.99 (1/100,000, maximum in 2005). The pattern of HFRS incidence showed a significant seasonality with an annual peak in spring from March to May (Fig 1, S2 Fig). The epidemic variability could be divided into three periods: period I (1998–2004), characterized by a high-level of HFRS incidence of over 17.6 (1/100,000) and a stable periodicity without limited intervention; period II (2005–2012), vaccination campaign and rat extermination program were implemented simultaneously and the local HFRS incidence decreased dramatically to a relatively low level at 10.0 (1/100,000); period III (2013–2015), the incidence of HFRS gradually rebounded. By using wavelet analysis, we found changes in endemic periodicity after 2006 (Fig 1B), possibly due to the rat extermination program and mass vaccination campaign that were conducted beginning in 2005. Here, to reduce the effect of human interventions on disease dynamics, we focused our analysis on the study period between 1998 and 2004 (period I). In period I, a total of 3914 HFRS cases were reported. The local rodent community mainly comprised Norway rat and the house mouse, with the former accounting for 94.52% of the total. The population density of the rodents ranged from 4.89% to 7.28%, with two relatively low values in January of 1998 and 1999.

### Causal relationships between environmental factors, Norway rat population density and HFRS transmission risk

We first examined the impact of land use and land cover changes on HFRS transmission. Land use changed very slightly in period I (S3 Fig), so the effect of habitat and land use change on rodent hosts and disease transmission were minimal during this time period. We then conducted a cross-correlation analysis of the climate variables, rodent population density, and HFRS incidence. The Norway rat population was found to be positively correlated with mean temperature (Pearson’s *r* = 0.23, *P* < 0.05), maximum temperature (*r* = 0.25, *P* < 0.05), minimum temperature (*r* = 0.23, *P* < 0.05), cumulative precipitation (*r* = 0.28, *P* < 0.05), and absolute humidity (*r* = 0.24, *P* < 0.05), with the maximum correlation coefficients occurring at 3, 3, 4, 4, and 4-month time lags, respectively (S4A Fig). HFRS incidence was strongly correlated with the climate variables. All the tested climate variables showed significant negative correlations with HFRS incidence, and maximum cross-correlations between temperature and HFRS incidence occurred at a lag of 3 months and other climate variables at a lag of 2 months (S4B Fig). Given the survival time of hantavirus outside the host and the incubation period for HFRS [51, 52], the longest time lag was set as 6 months.

Convergent cross mapping (CCM) was used to detect causality between these time series. However, rodent data had weak cross mapping skills for the climate variables. Only a marginally significant, weak causal effect was found between minimum temperature (with a 4-month lag, tp = -4) and absolute humidity (with a 3-month lag, tp = -3) on rodent population density, with cross map skills of 0.18 and 0.12, respectively (S5 Fig). To test the non-stationary (transient) correlation between Norway rat and climate variation, we conducted a further test of wavelet coherence analysis. The results showed scattered and small-sized distribution of significant areas with inconsistent phase differences (S6 Fig). In all, the combined results indicate that there might be a weak causal relationship between Norway rat and climate variables. Only relative humidity with a 1-month lag was identified as a significant causal factor for HFRS transmission (cross map skill, 0.86) by the CCM method (S7 Fig). The significance test distinguished anomalies of climate effects from shared seasonal cycle effects (S7B Fig).

### Model validation

**Fig 2:**
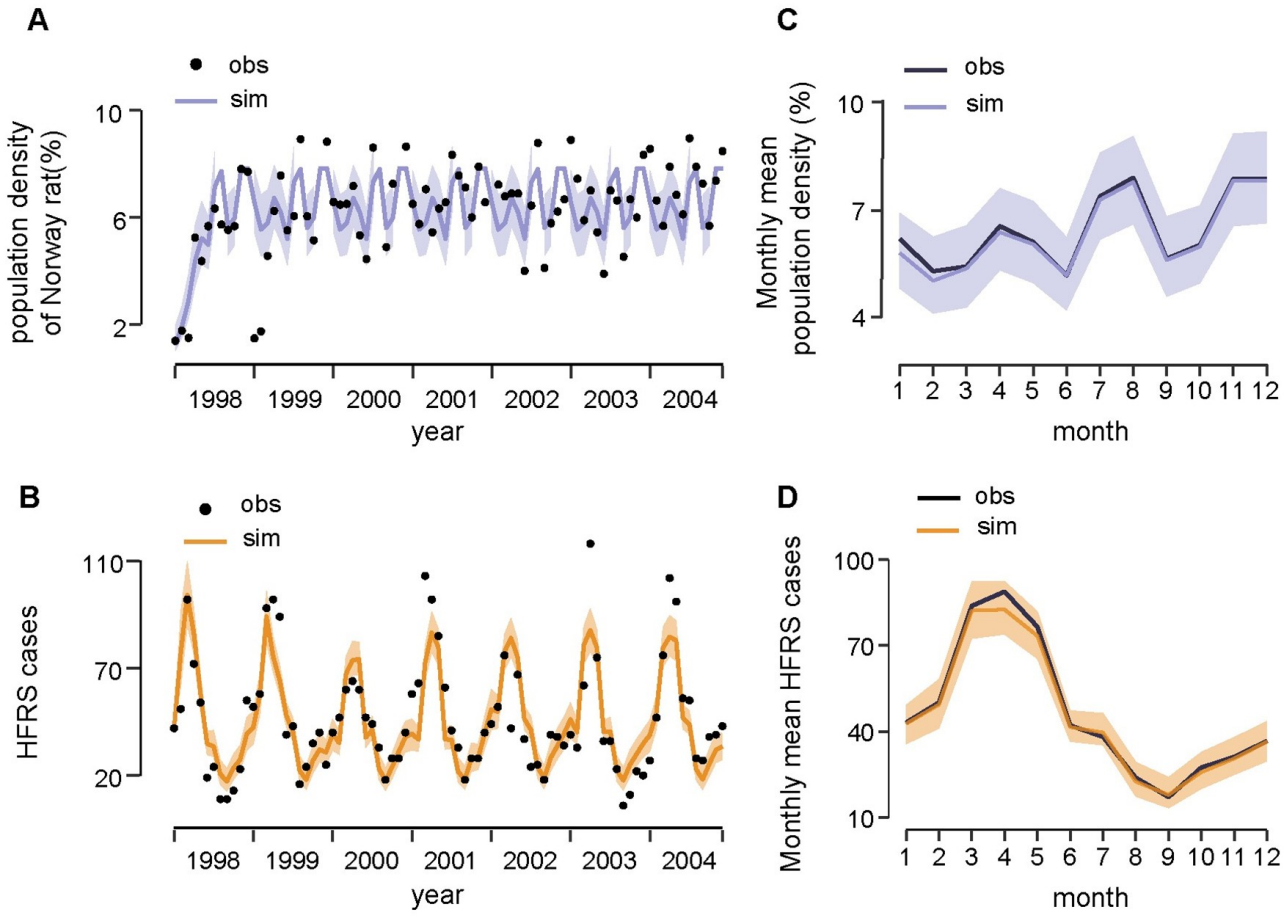
Fitting results of the dynamic model. (A) Time series of Norway rat population density and (B) HFRS cases. Black points indicate the real observations (obs) and lines indicate the simulated (sim) time series. Shaded areas indicate the 95% credible interval. (C) The season pattern of Norway rat population density and (D) HFRS cases. The 5% and 95% of simulated data are shown in shaded area.

To quantify the effect of climatic factors on rodent population density, we tested the models (see Materials and methods for details) with *θ*_*r*_ containing (*i*) minimum temperature, (*ii*) absolute humidity, (*iii*) both variables, and (*iv*) none of these climate variables. The goodness of fit for all the candidate models is provided in Table 1. The model without the climate influence was regarded as optimal to infer the population dynamics of Norway rat in a local urban setting (DIC = 9.17, R^2^ = 0.50, Fig 2A). The reproduction rates of the local Norway rat population, as estimated by our model, peaked in June, July, and October, and reached the lowest level in May and August (Table 2). The average life span of the Norway rat is estimated to be about 6 months, and the local environmental carrying capacity is estimated to be 8.24. The trace plot of Markov chains and the posterior distribution for the parameters in the optimal rodent model is shown in S8 Fig. All the parameter traces have passed the convergence test.

**Fig 3:**
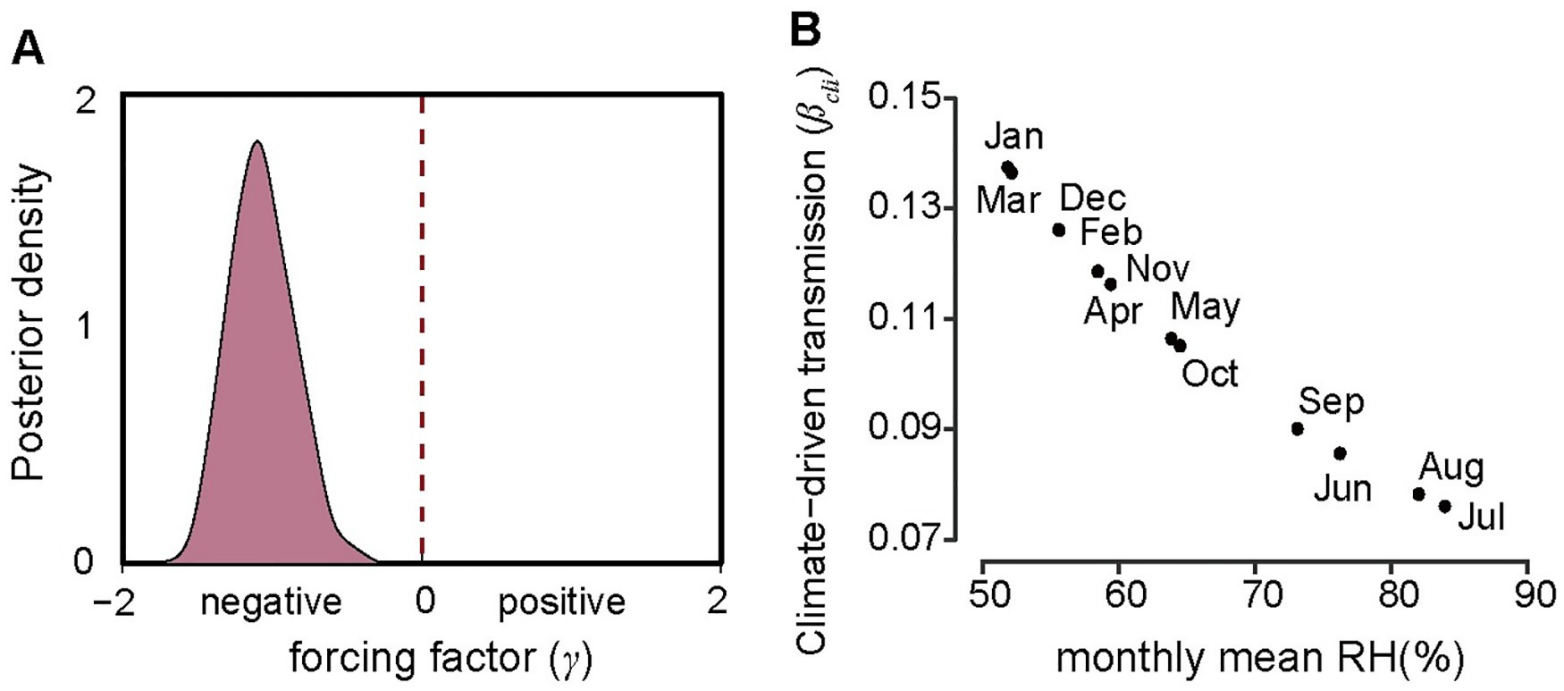
Force of infection and transmission rate of the seasonality of HFRS risk. (A) Posterior distribution of climate-driven transmission potential (*β*_*cli*_), represented by forcing factor (*γ*). The power function with negative exponent (*γ*) means a negative relationship between transmission and relative humidity. (B) The effect of seasonal changes in relative humidity on climate-driven transmission potential estimated from Eq (4).

**Fig 4:**
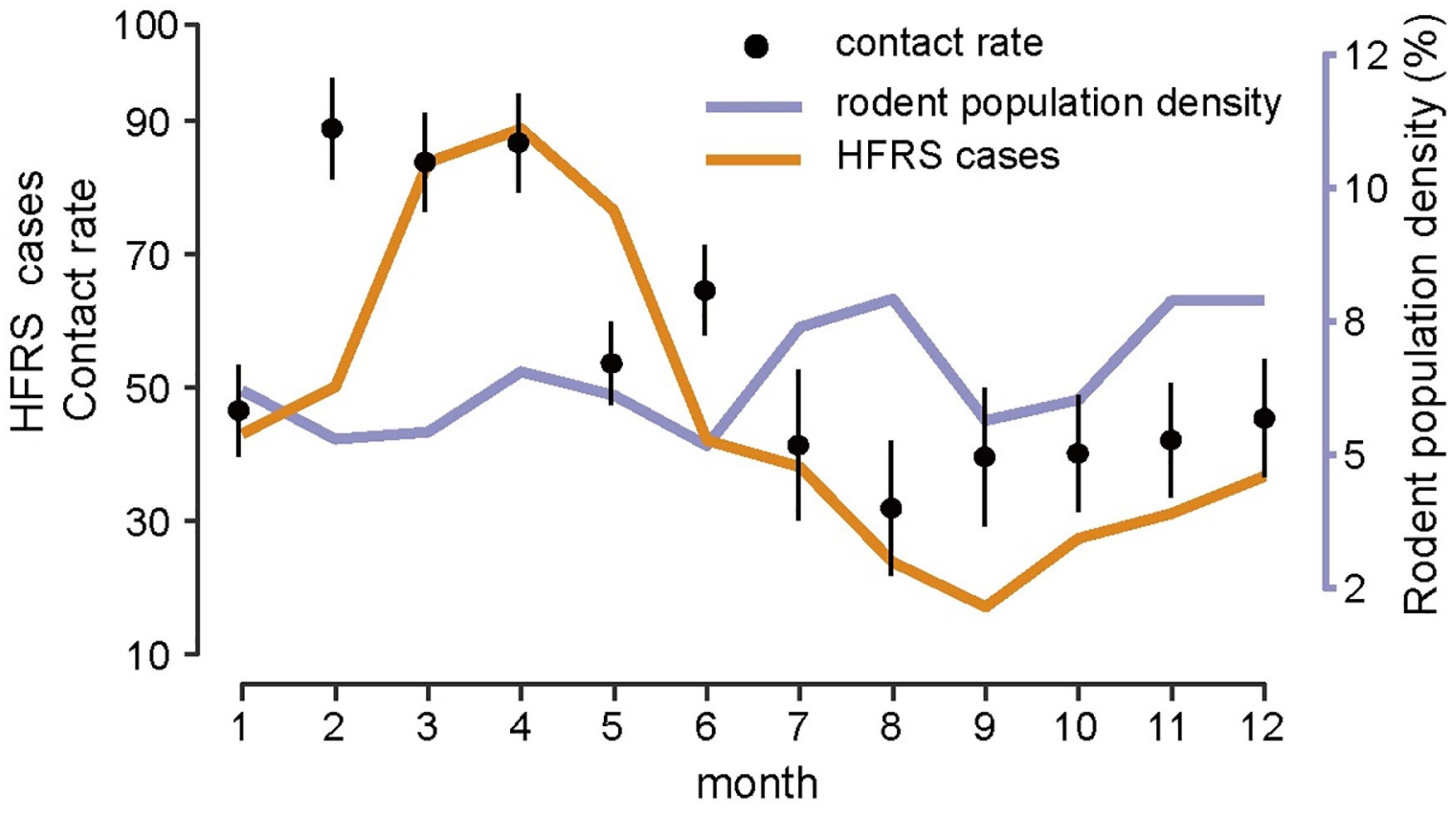
Season epidemics, rodent population dynamics and estimated contact rate. Error bars show the 95% credible intervals.

Based on the causal relationship tested by CCM, we constructed a HFRS dynamic model containing the infected rodent population and seasonal contact rate to quantify the specific effect of relative humidity on risk of HFRS (see Materials and methods for details). We constructed two models: one containing relative humidity and the other without. The model with relative humidity had a slightly better fit when the simulated HFRS cases correlated well with the observations and 75% of the variance was explained (R^2^ = 0.75, DIC = 5.57) (Fig 2B), relative to the model without relative humidity (R^2^ = 0.72, DIC = 7.89). The optimal models also captured the main seasonal patterns of both the rodent population density and reported cases (Fig 2C and 2D). The parameter *γ* in *β*_*cli*_ = *δ*_*3*_*RH γ t-1*, was estimated to be −1.23 (95% CI, -1.62– -0.83) (Fig 3A), indicating that relative humidity had a negative impact on HFRS transmission (Fig 3B). Additionally, during winter–spring, the estimated seasonal contact rates peaked from February to April (Fig 4), which may result from the behaviors of human and rodent. Most of the patients are farmers (77%) and it’s the slack time of winter-spring when people stay at home. Besides, due to the severe weather outside during that time, Norway rats would live and feed in closer proximity to human residence for favorable living conditions and available food, which could be a reason for the high contact rate. The trace plot of Markov chains and the posterior distribution for the parameters used in the optimal HFRS transmission model are shown in S9 Fig.

## Discussion

We used a mathematical model to quantify the impact of intrinsic and extrinsic factors on risk of HFRS, and to test the mechanistic understanding of HFRS transmission dynamics in a SEOV endemic area of China. By analyzing time-series data of Norway rat populations and SEOV infections, we found that the population dynamics of Norway rat in urban settings were relatively well-predicted by a simple model which excluded climatic conditions. Unusually, we demonstrated that a potential biotic driver (relative humidity) could enhance predictive ability of the HFRS transmission analysis.

Our results highlight the crucial role of mass immunization campaigns and rat extermination program in HFRS transmission control. It should be noted that SEOV infections began to rise again after 2013 in the post-vaccination era. In HFRS endemic areas where hosts are mainly wild rodents, fluctuations in the population density of wild rodents, such as striped field mouse or bank vole, are influenced by climatic factors and can mediate the effect of climate on the risk of HFRS transmission, mainly because of the close association between climate and host food supply [53]. However, in the SEOV-endemic area, the CCM results showed that the change in population density of the Norway rat was insensitive to climate variability. Our results differed from those of a previous study which found a relationship among the virus-carrying index, climate variables, and HFRS incidence by using structural equation modeling (SEM). This study reported that the Norway rat served as an important mediator of disease transmission when climate variability was found to influence the risk of HFRS [54]. This inconsistency may be due to a difference in sampling frequency as we surveyed on a monthly basis, while Guan et al. (2009) sampled quarterly. Additionally, the SEM analysis used a latent variable to account for measurement error but did not evaluate the contribution of specific variables to the transmission dynamics.

Norway rat is a domestic rodent whose lifestyle differs greatly from those of wild rodents. Norway rat lives in residential areas and feeds on various food items from humans instead of field crops [55]. Food resources and habitats are relatively stable and change only slightly with climate variability. Additionally, Norway rats breed throughout the year in Huludao City, where the winter temperatures rarely drop below −10 °C and residential areas have warmer conditions because of the winter heating policy in China. Winter breeding of wild rodents is limited because of low temperatures [56].

Our findings indicated that an abiotic factor, relative humidity, was a critical indicator of HFRS risk in the SEOV-endemic area of Huludao City, which is consistent with previous studies in which SEOV was the main virus type [57, 58]. We assumed that relative humidity may be associated with the survival or infectivity of SEOV in the environment, the activity of the rodent, and human hosts or the transmission process, although the underlying mechanism is not clear. We do not know of any relevant experiments on how environmental conditions affect survival of SEOV outside the host, while PUUV and HTNV have been confirmed to have longer infective periods at low temperatures and high humidities in experimental environments [59]. In our model, interannual variation in SEOV infections was partly explained by changes in relative humidity. A less intense outbreak in 2000 might be associated with a lower relative humidity-driven transmission potential (Fig 2B).

Some limitations in our study should be mentioned. First, rodent population density was extremely low in the springs of 1998 and 1999, which reduced the overall explanatory ability of our model. Second, prevalence of SEOV infection in Norway rats was assessed by mathematical model due to lack of available data. Third, to ameliorate the influence of vaccination campaign and other interventions, only the selected time range of data was used to detect the relationship between intrinsic/extrinsic drivers and SEOV transmission. Further efforts should be made to improve model reliability through efforts such as addition of data to the available dataset. More attention should be paid to the association between economic development, such as infrastructure improvement, the spatial distribution of Norway rat, and induced SEOV infections across districts in subsequent studies.

The SEOV-related HFRS is posing a health threat to an increasing number of people. Although the Norway rat is found everywhere, SEOV cases are not. We suspect this may mainly be due to increased surveillance and attention focused on this pathogen, as SEOV previously caused many asymptomatic human infections previously. In conclusion, based on the longitudinal and complete dataset, our study yielded a proper framework for understanding the intrinsic transmission dynamics and extrinsic effects on HFRS risk caused by SEOV, especially at the human-animal-environment interface. The framework can be flexibly adjusted when more information is available. Furthermore, we provided clues to potential environmental drivers on SEOV transmission dynamics, which would be useful for further research related to public health issues.

## Supporting information

S1 Fig The geographical location of Huludao City. (A) Huludao City (orange) is located in northwest of Liaoning Province, adjacent to the Bohai sea. (B) Liaoning Province is highlighted in red in China map.
(TIF)

S2 Fig Seasonal distribution of HFRS cases over three periods. (TIF)

S3 Fig Land cover composition in Huludao City from 1998 to 2015. The land cover classification is according to IPCC land categories. Other includes shrubland, sparse vegetation, bare area and water.
(TIF)

S4 Fig The relationship between hemorrhagic fever with renal syndrome (HFRS) incidence, Norway rat population density and environmental variables with time lags. (A) Cross-correlations between the population density of Norway rats and the climate variables. (B) Cross-correlations between HFRS and climate variables. TM, monthly mean temperature; TMAX, monthly mean maximum temperature; TMIN, monthly mean minimum temperature; PREC, monthly cumulative precipitation; RH, monthly mean relative humidity; AH, monthly mean absolutely humidity; RN.(t), Norway rat population density at time t; IN.(t), HFRS incidence at time t.
(TIF)

S5 Fig The causal relationship between climate variables and rodent host population, and between climate variables and HFRS incidence. (A) The convergent cross mapping results for Norway rat population density and climate variables with time lags (tp) and for HFRS incidence (B). The causal relationship was regarded as significant (P < 0.05) when the solid line (cross map skill of the observed data) exceeded the dashed line (surrogate data). TM, monthly mean temperature; TMAX, monthly mean maximum temperature; TMIN, monthly mean minimum temperature; PREC, monthly cumulative precipitation; RH, monthly mean relative humidity; AH, monthly mean absolutely humidity.
(TIF)

S6 Fig Wavelet coherence between Norway rat population density and climate variables from 1998 to 2004. Arrows showed phase differences between Norway rat population density and climate variables. The straight down represents climate variables lead Norway rat population density 1/4 period. Legend is wavelet coherence level. The parts inside white contour shows a significant relationship. The parts outside the transparent cone have affected by edge effect. TM, monthly mean temperature; TMAX, monthly mean maximum temperature; TMIN, monthly mean minimum temperature; PREC, monthly cumulative precipitation; RH, monthly mean relative humidity; AH, monthly mean absolutely humidity; RN, Norway rat population density.
(TIF)

S7 Fig Detecting cross-map causality beyond shared seasonality of environmental drivers on hemorrhagic fever with renal syndrome. (A) Time series of hemorrhagic fever with renal syndrome (HFRS) incidence (orange) and relative humidity (green). (B) The convergent cross mapping results at different time series length for HFRS incidence and relative humidity (RH) and surrogate RH (surr_RH) with 1-month lag (tp = -1). The causal relationship was regarded as significant (P < 0.05) when the solid line (cross map skill of the observed data) exceeded the dashed line (surrogate data).
(TIF)

S8 Fig Estimated parameters in Norway rat population dynamic model. (A) The trace plot of markov chains of the parameters after 1 million burn-in. (B) The posterior distribution of the parameters after 1 million burn-in. All the estimations have passed the Gelman-Rubin-Brooks MCMC convergence diagnostic.
(TIF)

S9 Fig Estimated parameters in HFRS transmission dynamic model. (A) The trace plot of markov chains of the parameters after 1 million burn-in. (B) The posterior distribution of the parameters after 1 million burn-in. All the estimations have passed the Gelman-Rubin-Brooks MCMC convergence diagnostic.
(TIF)

## References

[1] Kariwa H, Yoshimatsu K, Arikawa J. Hantavirus infection in east Asia. Comp Immunol Microbiol Infect Dis. 2007;30(5–6): 341–56. 10.1016/j.cimid.2007.05.011 17655929

[2] Zhang Y-Z, Zou Y, Fu ZF, Plyusnin A. Hantavirus infections in humans and animals, China. Emerg Infect Dis. 2010;16(8): 1195 10.3201/eid1608.090470 20678311 PMC3298307

[3] Plyusnin A, Vapalahti O, Vaheri A. Hantaviruses: genome structure, expression and evolution. J Gen Virol. 1996;77(11): 2677–87. 10.1099/0022-1317-77-11-2677 8922460

[4] Plyusnin A, Morzunov S. Virus evolution and genetic diversity of hantaviruses and their rodent hosts. Hantaviruses: Springer Berlin Heidelberg; 2001 pp. 47–75. 10.1007/978-3-642-56753-7_4 11217406

[5] Tian H, Hu S, Cazelles B, Chowell G, Gao L, Laine M, et al Urbanization prolongs hantavirus epidemics in cities. Proc Natl Acad Sci USA. 2018;115(18): 4707–12. 10.1073/pnas.1712767115 29666240 PMC5939059

[6] Metcalf CJE, Walter KS, Wesolowski A, Buckee CO, Shevliakova E, Tatem AJ, et al Identifying climate drivers of infectious disease dynamics: recent advances and challenges ahead. Proc Biol Sci. 2017;284(1860). 10.1098/rspb.2017.0901 28814655 PMC5563806

[7] Roda Gracia J, Schumann B, Seidler A. Climate Variability and the Occurrence of Human Puumala Hantavirus Infections in Europe: A Systematic Review. Zoonoses Public Health. 2015;62(6): 465–78. 10.1111/zph.12175 25557350

[8] Tian H, Stenseth NC. The ecological dynamics of hantavirus diseases: From environmental variability to disease prevention largely based on data from China. PLoS Negl Trop Dis. 2019;13(2): e0006901 10.1371/journal.pntd.0006901 30789905 PMC6383869

[9] Tian HY, Yu PB, Luis AD, Bi P, Cazelles B, Laine M, et al Changes in rodent abundance and weather conditions potentially drive hemorrhagic fever with renal syndrome outbreaks in Xi’an, China, 2005–2012. PLoS Negl Trop Dis. 2015;9(3): e0003530 10.1371/journal.pntd.0003530 25822936 PMC4378853

[10] Clement J, Maes P, Van Ypersele de Strihou C, van der Groen G, Barrios JM, Verstraeten WW, et al Beechnuts and outbreaks of nephropathia epidemica (NE): of mast, mice and men. Nephrol Dial Transplant. 2010;25(6): 1740–6. 10.1093/ndt/gfq122 20237057

[11] Yates TL, Mills JN, Parmenter CA, Ksiazek TG, Parmenter RR, Vande Castle JR, et al The ecology and evolutionary history of an emergent disease: hantavirus pulmonary syndrome: evidence from two El Niño episodes in the American southwest suggests that El Niño–driven precipitation, the initial catalyst of a trophic cascade that results in a delayed density-dependent rodent response, is sufficient to predict heightened risk for human contraction of hantavirus pulmonary syndrome. Biosci. 2002;52(11): 989–98.

[12] Clement J, Vercauteren J, Verstraeten WW, Ducoffre G, Barrios JM, Vandamme AM, et al Relating increasing hantavirus incidences to the changing climate: the mast connection. Int J Health Geogr. 2009;8(1): 1 10.1186/1476-072x-8-1 19149870 PMC2642778

[13] Piechotowski I, Brockmann SO, Schwarz C, Winter CH, Ranft U, Pfaff G. Emergence of hantavirus in South Germany: rodents, climate and human infections. Parasitol Res. 2008;103 Suppl 1: S131–7. 10.1007/s00436-008-1055-8 19030895

[14] Tian H, Yu P, Cazelles B, Xu L, Tan H, Yang J, et al Interannual cycles of Hantaan virus outbreaks at the human-animal interface in Central China are controlled by temperature and rainfall. Proc Natl Acad Sci USA. 2017;114(30): 8041–6. 10.1073/pnas.1701777114 28696305 PMC5544290

[15] Himsworth CG, Parsons KL, Jardine C, Patrick DM. Rats, cities, people, and pathogens: a systematic review and narrative synthesis of literature regarding the ecology of rat-associated zoonoses in urban centers. Vector Borne Zoonotic Dis. 2013;13(6): 349–59. 10.1089/vbz.2012.1195 23590323

[16] Clement J, LeDuc JW, Lloyd G, Reynes J-M, McElhinney L, Van Ranst M, et al Wild Rats, Laboratory Rats, Pet Rats: Global Seoul Hantavirus Disease Revisited. Viruses. 2019;11(7): 652 10.3390/v11070652 31319534 PMC6669632

[17] Costa F, Porter FH, Rodrigues G, Farias H, de Faria MT, Wunder EA, et al Infections by Leptospira interrogans, Seoul virus, and Bartonella spp. among Norway rats (Rattus norvegicus) from the urban slum environment in Brazil. Vector Borne Zoonotic Dis. 2014;14(1): 33–40. 10.1089/vbz.2013.1378 24359425 PMC3880909

[18] Song G. Epidemiological progresses of hemorrhagic fever with renal syndrome in China. Chin Med J (Engl). 1999;112(5): 472–7. 11593522

[19] Jameson L, Logue C, Atkinson B, Baker N, Galbraith S, Carroll M, et al The continued emergence of hantaviruses: isolation of a Seoul virus implicated in human disease, United Kingdom, October 2012. Euro Surveill. 2013;18(1): 20344 23305714

[20] Heyman P, Plyusnina A, Berny P, Cochez C, Artois M, Zizi M, et al Seoul hantavirus in Europe: first demonstration of the virus genome in wild Rattus norvegicus captured in France. Eur J Clin Microbiol Infect Dis. 2004;23(9): 711–7. 10.1007/s10096-004-1196-3 15322934

[21] Kerins JL, Koske SE, Kazmierczak J, Austin C, Gowdy K, Dibernardo A. Outbreak of Seoul virus among rats and rat owners—United States and Canada, 2017. Canada communicable disease report = Releve des maladies transmissibles au Canada. 2018;44(2): 71–4. 10.14745/ccdr.v44i02a07 29770103 PMC5864277

[22] Wang H, Yoshimatsu K, Ebihara H, Ogino M, Araki K, Kariwa H, et al Genetic diversity of hantaviruses isolated in China and characterization of novel hantaviruses isolated from Niviventer confucianus and Rattus rattus. Virology. 2000;278(2): 332–45. 10.1006/viro.2000.0630 11118357

[23] Zhang YZ, Zou Y, Fu ZF, Plyusnin A. Hantavirus infections in humans and animals, China. Emerg Infect Dis. 2010;16(8): 1195 10.3201/eid1608.090470 20678311 PMC3298307

[24] Zhang YZ, Zhang FX, Gao N, Wang JB, Zhao ZW, Li MH, et al Hantaviruses in rodents and humans, inner Mongolia Autonomous region, China. Emerg Infect Dis. 2009;15(6): 885 10.3201/eid1506.081126 19523286 PMC2727351

[25] Lin XD, Guo WP, Wang W, Zou Y, Hao ZY, Zhou DJ, et al Migration of Norway rats resulted in the worldwide distribution of Seoul hantavirus today. J Virol. 2012;86(2): 972–81. 10.1128/JVI.00725-11 22090114 PMC3255798

[26] Yan L, Fang L-Q, Huang H-G, Zhang L-Q, Feng D, Zhao W-J, et al Landscape elements and Hantaan virus–related hemorrhagic fever with renal syndrome, People’s Republic of China. Emerg Infect Dis. 2007;13(9): 1301 10.3201/eid1309.061481 18252099 PMC2857277

[27] Kim WK, No JS, Lee SH, Song DH, Lee D, Kim JA, et al Multiplex PCR-based next-generation sequencing and global diversity of seoul virus in humans and rats. Emerg Infect Dis. 2018;24(2): 249 10.3201/eid2402.171216 29350137 PMC5782898

[28] Clement J, LeDuc JW, McElhinney LM, Reynes J-M, Van Ranst M, Calisher CH. Clinical characteristics of ratborne seoul hantavirus disease. Emerg Infect Dis. 2019;25(2): 387 10.3201/eid2502.181643 30666956 PMC6346471

[29] Lixin Chen. Epidemiological survey of hemorrhagic fever with renal syndrome in Huludao City from 1992 to 2001. Chinese Journal of Vector Biology and Control. 2004;15(6): 465–6.

[30] WR G, TF s. Epidemiological analysis of Hemorrhagic with Renal Syndrome in Huludao city from 1997–2001. China J Vector Biology & control. 2003;14: 145–6.

[31] Lei C, Hongbo Y, Ping W. Effect evaluation of intervention measures for hemorrhagic fever with renal syndrome in Shenyang. Strait Journal of Preventive Medicine. 2014;3: 34–5.

[32] Xiao H, Tian HY, Cazelles B, Li XJ, Tong SL, Gao LD, et al Atmospheric moisture variability and transmission of hemorrhagic fever with renal syndrome in Changsha City, Mainland China, 1991–2010. PLoS Negl Trop Dis. 2013;7(6): 2260 10.1371/journal.pntd.0002260 23755316 PMC3674989

[33] Zhang YZ, Dong X, Li X, Ma C, Xiong HP, Yan GJ, et al Seoul virus and hantavirus disease, Shenyang, People’s Republic of China. Emerg Infect Dis. 2009;15(2): 200–6. 10.3201/eid1502.080291 19193263 PMC2662651

[34] Zhang X, Guo YP, B L. Surveillance on mice in HFRS epidemic focus, Huludao city. China J Vector Biology & control. 2006;17: 142–3.

[35] Cazelles B, Chavez M, Berteaux D, Menard F, Vik JO, Jenouvrier S, et al Wavelet analysis of ecological time series. Oecologia. 2008;156(2): 287–304. 10.1007/s00442-008-0993-2 18322705

[36] Cazelles B. Symbolic dynamics for identifying similarity between rhythms of ecological time series. Ecol Lett. 2004;7(9): 755–63. 10.1111/j.1461-0248.2004.00629.x

[37] Sugihara G. Nonlinear forecasting for the classification of natural time series. Philosophical Transactions of the Royal Society of London Series A: Physical and Engineering Sciences. 1994;348(1688): 477–95.

[38] Sugihara G, May RM. Nonlinear forecasting as a way of distinguishing chaos from measurement error in time series. Nature. 1990;344(6268): 734 10.1038/344734a0 2330029

[39] Sugihara G, May R, Ye H, Hsieh C-h, Deyle E, Fogarty M, et al Detecting causality in complex ecosystems. science. 2012;338(6106): 496–500. 10.1126/science.1227079 22997134

[40] Sugihara G, May RM. Nonlinear forecasting as a way of distinguishing chaos from measurement error in time series. Nature. 1990;344(6268): 734–41. 10.1038/344734a0 2330029

[41] Ye H, Deyle ER, Gilarranz LJ, Sugihara G. Distinguishing time-delayed causal interactions using convergent cross mapping. Sci Rep. 2015;5: 14750 10.1038/srep14750 26435402 PMC4592974

[42] Deyle ER, Maher MC, Hernandez RD, Basu S, Sugihara G. Global environmental drivers of influenza. Proc Natl Acad Sci U S A. 2016;113(46): 13081–6. 10.1073/pnas.1607747113 27799563 PMC5135382

[43] Zhou J, Hethcote HW. Population size dependent incidence in models for diseases without immunity. J Math Biol. 1994;32(8): 809–34. 10.1007/bf00168799 7814995

[44] Verhulst P-F. Notice sur la loi que la population suit dans son accroissement. Corresp Math Phys. 1838;10: 113–26.

[45] Decai Y. Analysis on the fecundity of the populations of brown rat and small rat in Dalian port. Chinese Journal of Pest Control. 1991;7(4): 272–4.

[46] Davis DE. The characteristics of rat populations. Q Rev Biol. 1953;28(4): 373–401. 10.1086/399860 13121239

[47] Glass GE, Childs JE, Korch GW, LeDuc JW. Association of intraspecific wounding with hantaviral infection in wild rats (Rattus norvegicus). Epidemiol Infect. 1988;101(2): 459–72. 10.1017/s0950268800054418 3141203 PMC2249393

[48] Childs JE, Glass GE, Korch GW, LeDuc JW. Effects of hantaviral infection on survival, growth and fertility in wild rat (Rattus norvegicus) populations of Baltimore, Maryland. Journal of Wildlife Diseases. 1989;25(4): 469–76. 10.7589/0090-3558-25.4.469 2572705

[49] Haario H, Laine M, Mira A, Saksman E. DRAM: efficient adaptive MCMC. Stat comput. 2006;16(4): 339–54. 10.1007/s11222-006-9438-0

[50] Cowles MK, Carlin BP. Markov chain Monte Carlo convergence diagnostics: a comparative review. J Am Stat Assoc. 1996;91(434): 883–904. 10.2307/2291683

[51] Hardestam J, Simon M, Hedlund K, Vaheri A, Klingström J, Lundkvist Å. Ex vivo stability of the rodent-borne Hantaan virus in comparison to that of arthropod-borne members of the Bunyaviridae family. Appl Environ Microbiol. 2007;73(8): 2547–51. 10.1128/AEM.02869-06 17337567 PMC1855600

[52] Young JC, Hansen GR, Graves TK, Deasy MP, Humphreys JG, Fritz CL, et al The incubation period of hantavirus pulmonary syndrome. Am J Trop Med Hyg. 2000;62(6): 714–7. 10.4269/ajtmh.2000.62.714 11304061

[53] Makary P, Kanerva M, Ollgren J, Virtanen MJ, Vapalahti O, Lyytikainen O. Disease burden of Puumala virus infections, 1995–2008. Epidemiol Infect. 2010;138(10): 1484–92. 10.1017/S0950268810000087 20109263

[54] Guan P, Huang D, He M, Shen T, Guo J, Zhou B. Investigating the effects of climatic variables and reservoir on the incidence of hemorrhagic fever with renal syndrome in Huludao City, China: a 17-year data analysis based on structure equation model. BMC Infect Dis. 2009;9(1): 109 10.1186/1471-2334-9-109 19583875 PMC2720978

[55] Feng AY, Himsworth CG. The secret life of the city rat: a review of the ecology of urban Norway and black rats (Rattus norvegicus and Rattus rattus). Urban Ecosystems. 2014;17(1): 149–62. 10.1007/s11252-013-0305-4

[56] Smyth M. Winter breeding in woodland mice, Apodemus sylvaticus, and voles, Clethrionomys glareolus and Microtus agrestis, near Oxford. J Anim Ecol. 1966;35(3): 471–85. 10.2307/2486

[57] Liu X, Jiang B, Gu W, Liu Q. Temporal trend and climate factors of hemorrhagic fever with renal syndrome epidemic in Shenyang City, China. BMC Infect Dis. 2011;11(1): 331 10.1186/1471-2334-11-331 22133347 PMC3247297

[58] Fang LQ, Wang XJ, Liang S, Li YL, Song SX, Zhang WY, et al Spatiotemporal trends and climatic factors of hemorrhagic fever with renal syndrome epidemic in Shandong Province, China. PLoS Negl Trop Dis. 2010;4(8): e789 10.1371/journal.pntd.0000789 20706629 PMC2919379

[59] Kallio ER, Klingstrom J, Gustafsson E, Manni T, Vaheri A, Henttonen H, et al Prolonged survival of Puumala hantavirus outside the host: evidence for indirect transmission via the environment. J Gen Virol. 2006;87(8): 2127–34. 10.1099/vir.0.81643-0 16847107

