# Supplementary figures and images for: Intrinsic and extrinsic drivers of transmission dynamics of hemorrhagic fever with renal syndrome caused by Seoul hantavirus
Source: PLoS Negl Trop Dis. 2019 Sep 23;13(9):e0007757. doi: 10.1371/journal.pntd.0007757 (PMC6776365; doi:10.1371/journal.pntd.0007757)

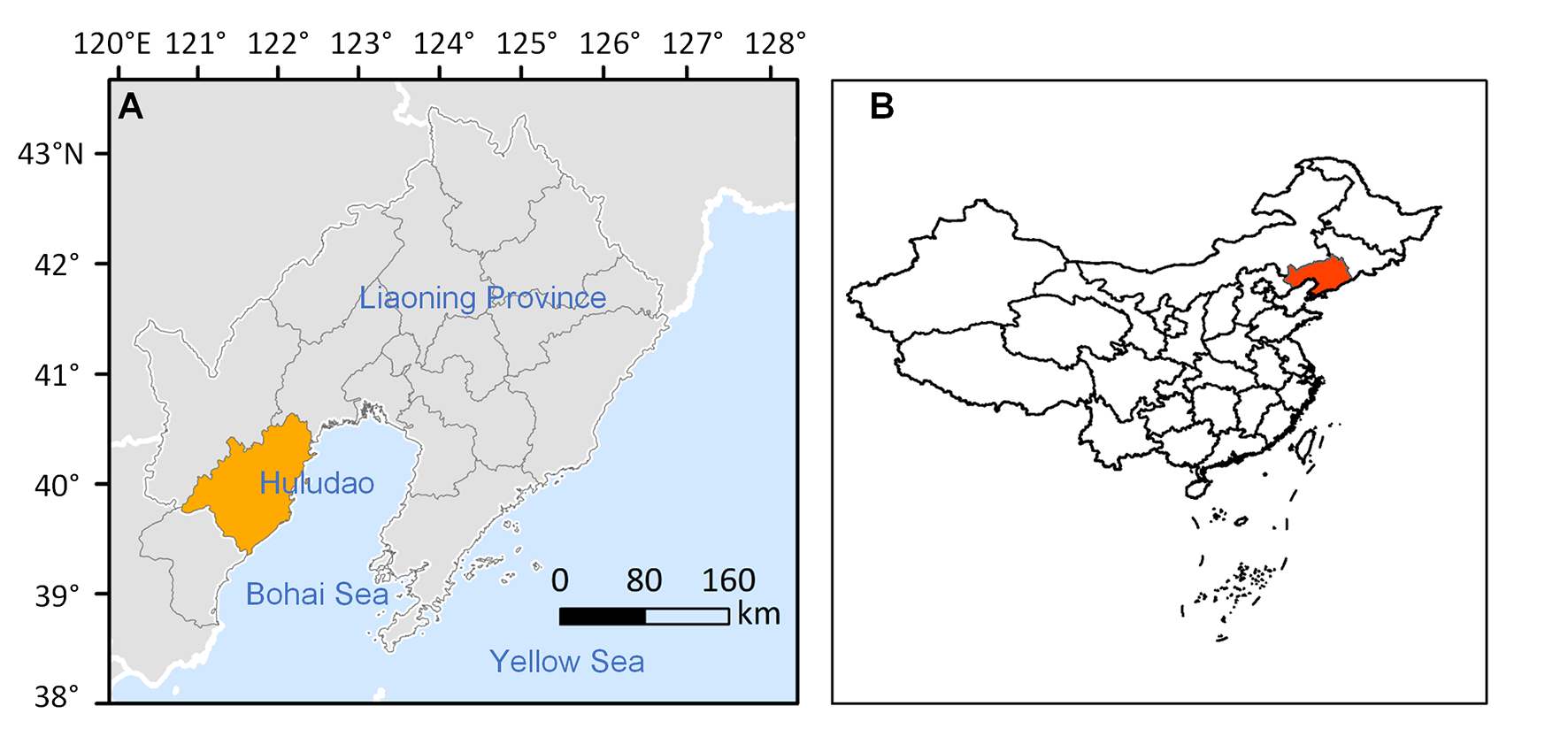

Supplement: S1 Fig — (A) Huludao City (orange) is located in northwest of Liaoning Province, adjacent to the Bohai sea. (B) Liaoning Province is highlighted in red in China map. (TIF) [file pntd.0007757.s001.tif]

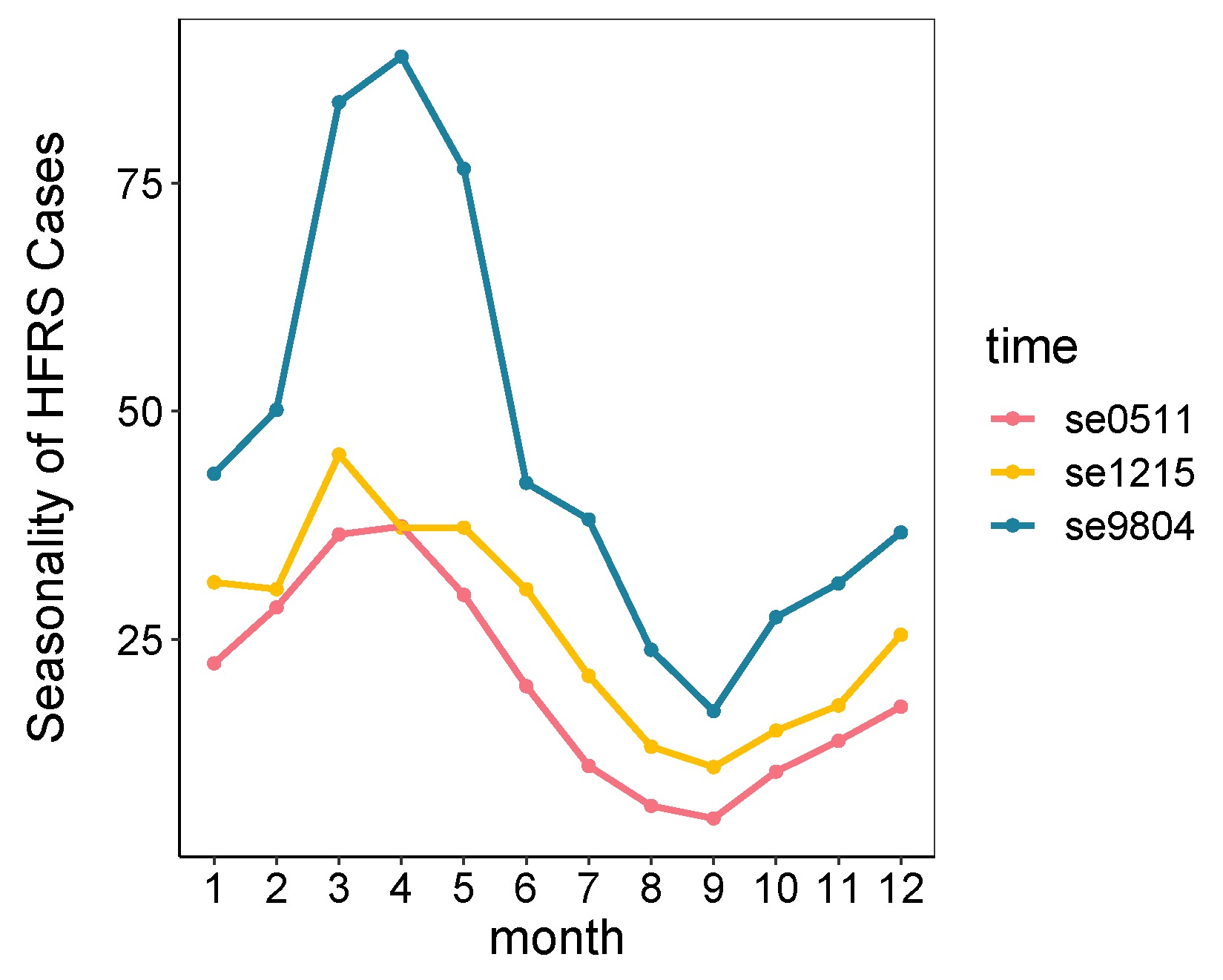

Supplement: S2 Fig — (TIF) [file pntd.0007757.s002.tif]

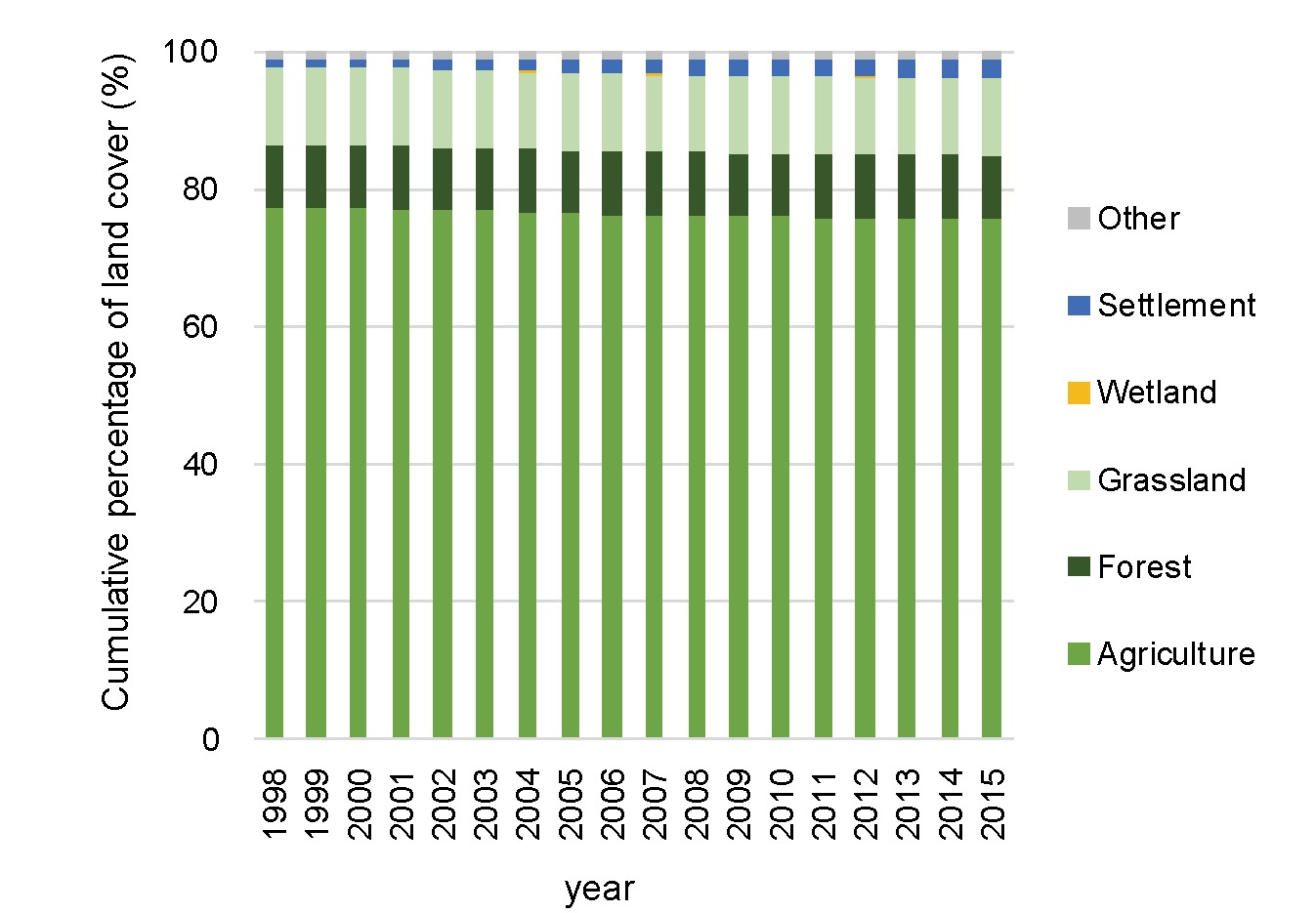

Supplement: S3 Fig — The land cover classification is according to IPCC land categories. Other includes shrubland, sparse vegetation, bare area and water. (TIF) [file pntd.0007757.s003.tif]

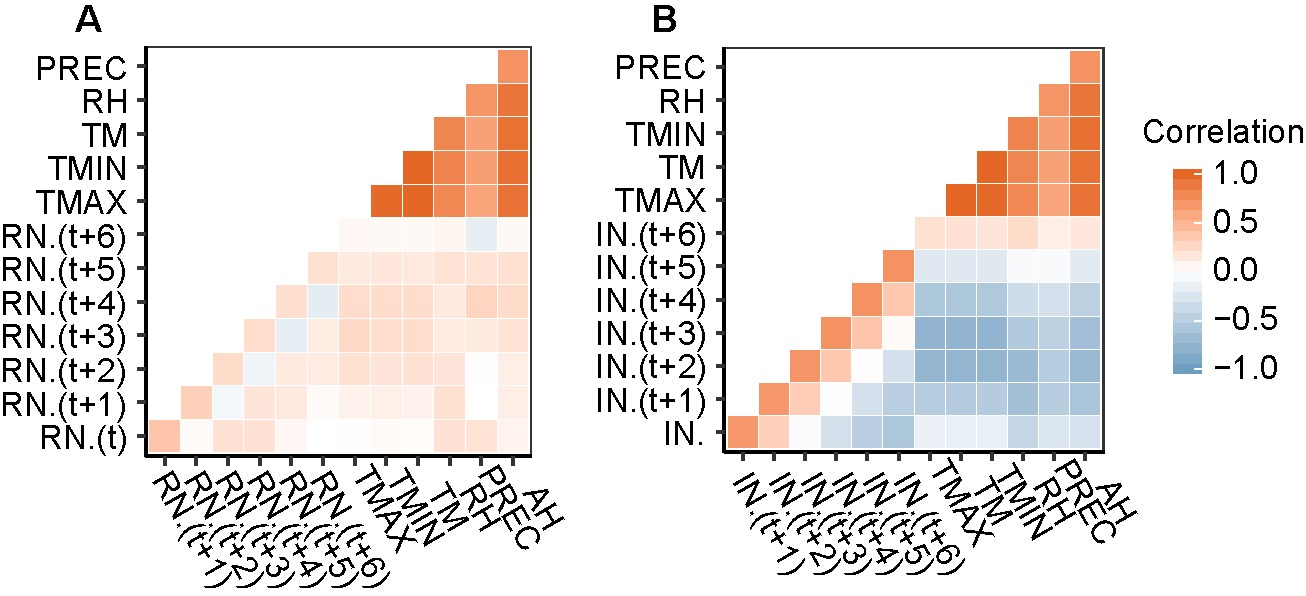

Supplement: S4 Fig — (A) Cross-correlations between the population density of Norway rats and the climate variables. (B) Cross-correlations between HFRS and climate variables. TM, monthly mean temperature; TMAX, monthly mean maximum temperature; TMIN, monthly mean minimum temperature; PREC, monthly cumulative precipitation; RH, monthly mean relative humidity; AH, monthly mean absolutely humidity; RN.(t), Norway rat population density at time t; IN.(t), HFRS incidence at time t. (TIF) [file pntd.0007757.s004.tif]

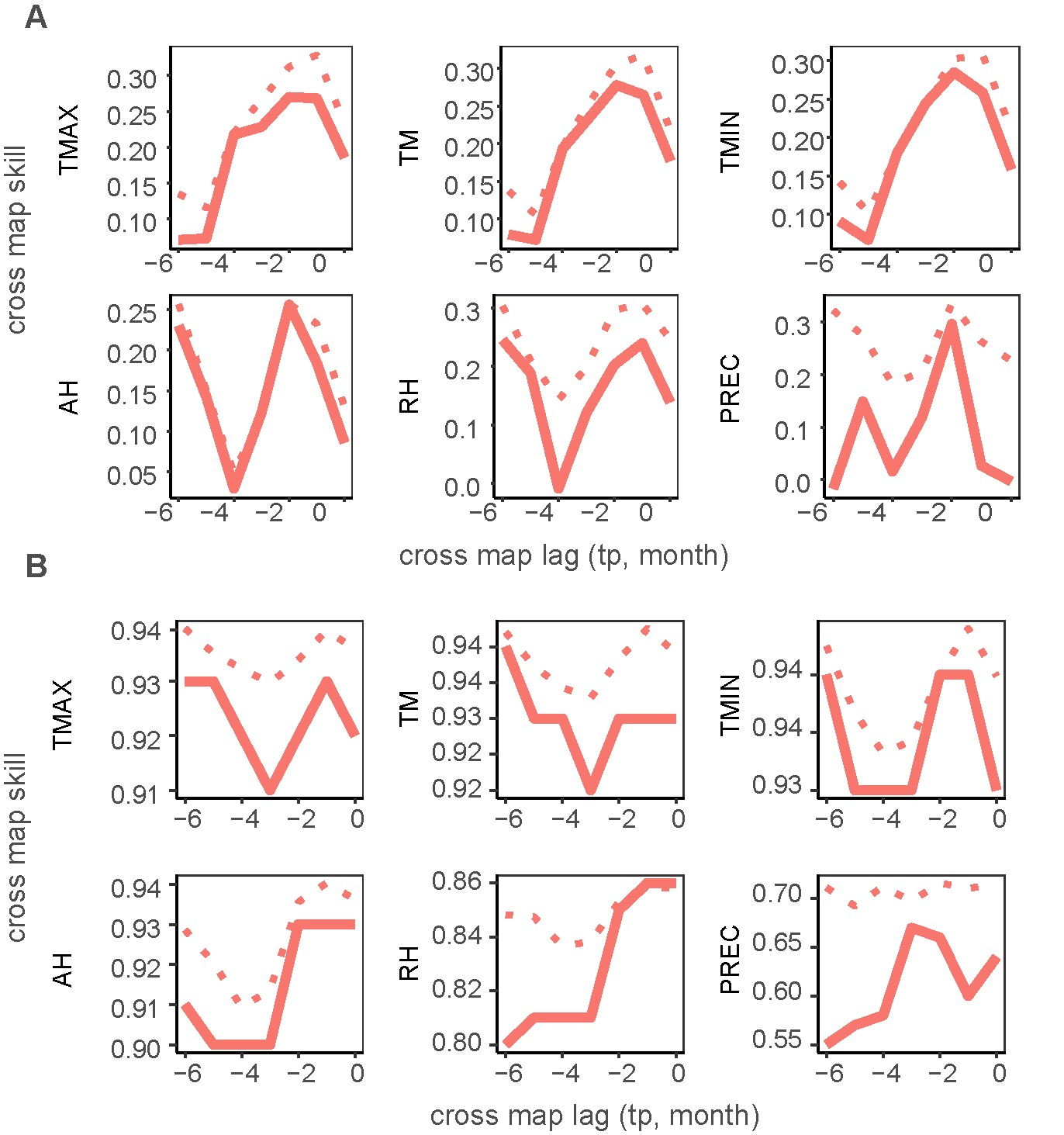

Supplement: S5 Fig — (A) The convergent cross mapping results for Norway rat population density and climate variables with time lags (tp) and for HFRS incidence (B). The causal relationship was regarded as significant (P < 0.05) when the solid line (cross map skill of the observed data) exceeded the dashed line (surrogate data). TM, monthly mean temperature; TMAX, monthly mean maximum temperature; TMIN, monthly mean minimum temperature; PREC, monthly cumulative precipitation; RH, monthly mean relative humidity; AH, monthly mean absolutely humidity. (TIF) [file pntd.0007757.s005.tif]

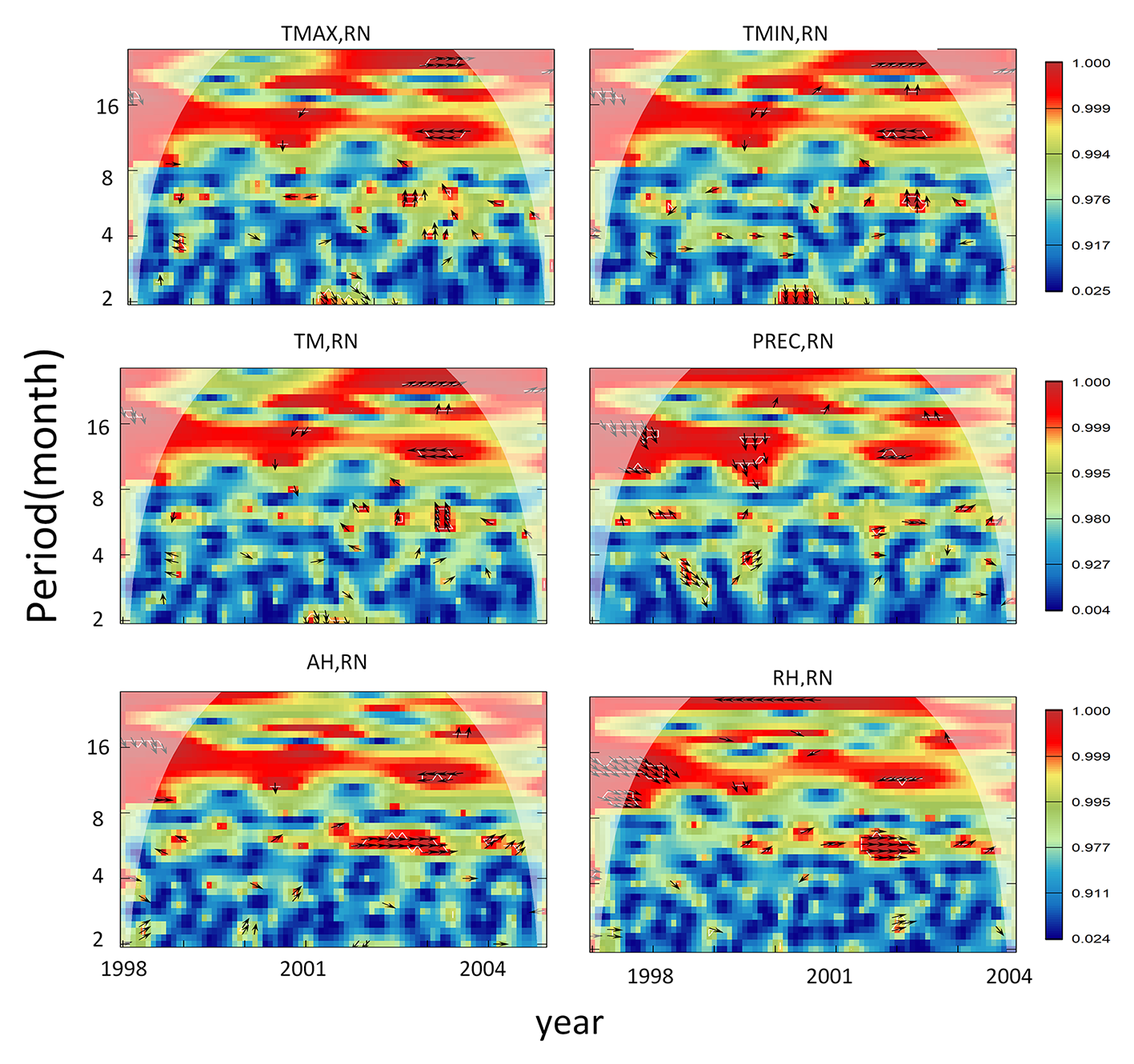

Supplement: S6 Fig — Arrows showed phase differences between Norway rat population density and climate variables. The straight down represents climate variables lead Norway rat population density 1/4 period. Legend is wavelet coherence level. The parts inside white contour shows a significant relationship. The parts outside the transparent cone have affected by edge effect. TM, monthly mean temperature; TMAX, monthly mean maximum temperature; TMIN, monthly mean minimum temperature; PREC, monthly cumulative precipitation; RH, monthly mean relative humidity; AH, monthly mean absolutely humidity; RN, Norway rat population density. (TIF) [file pntd.0007757.s006.tif]

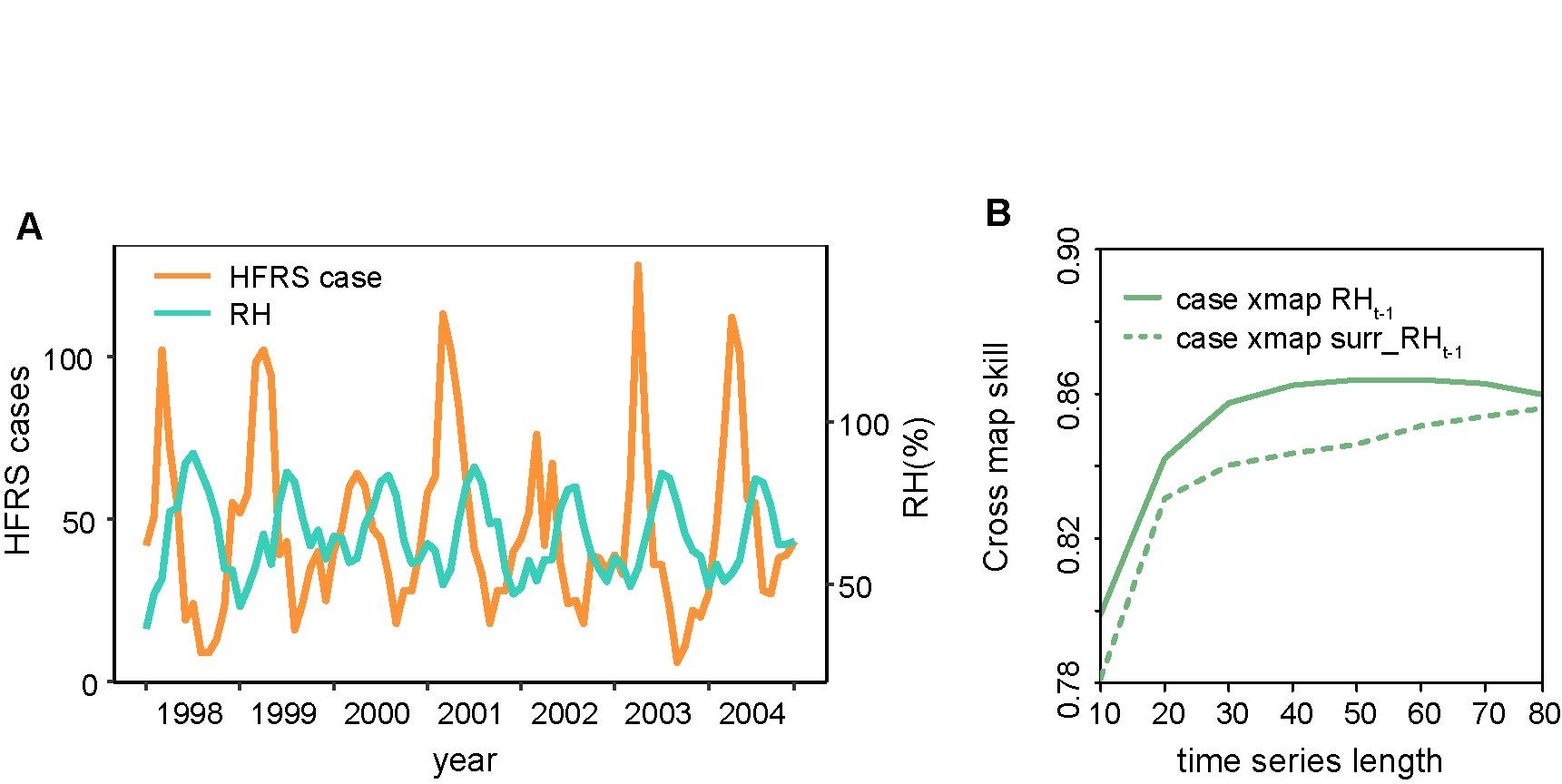

Supplement: S7 Fig — (A) Time series of hemorrhagic fever with renal syndrome (HFRS) incidence (orange) and relative humidity (green). (B) The convergent cross mapping results at different time series length for HFRS incidence and relative humidity (RH) and surrogate RH (surr_RH) with 1-month lag (tp = -1). The causal relationship was regarded as significant (P < 0.05) when the solid line (cross map skill of the observed data) exceeded the dashed line (surrogate data). (TIF) [file pntd.0007757.s007.tif]

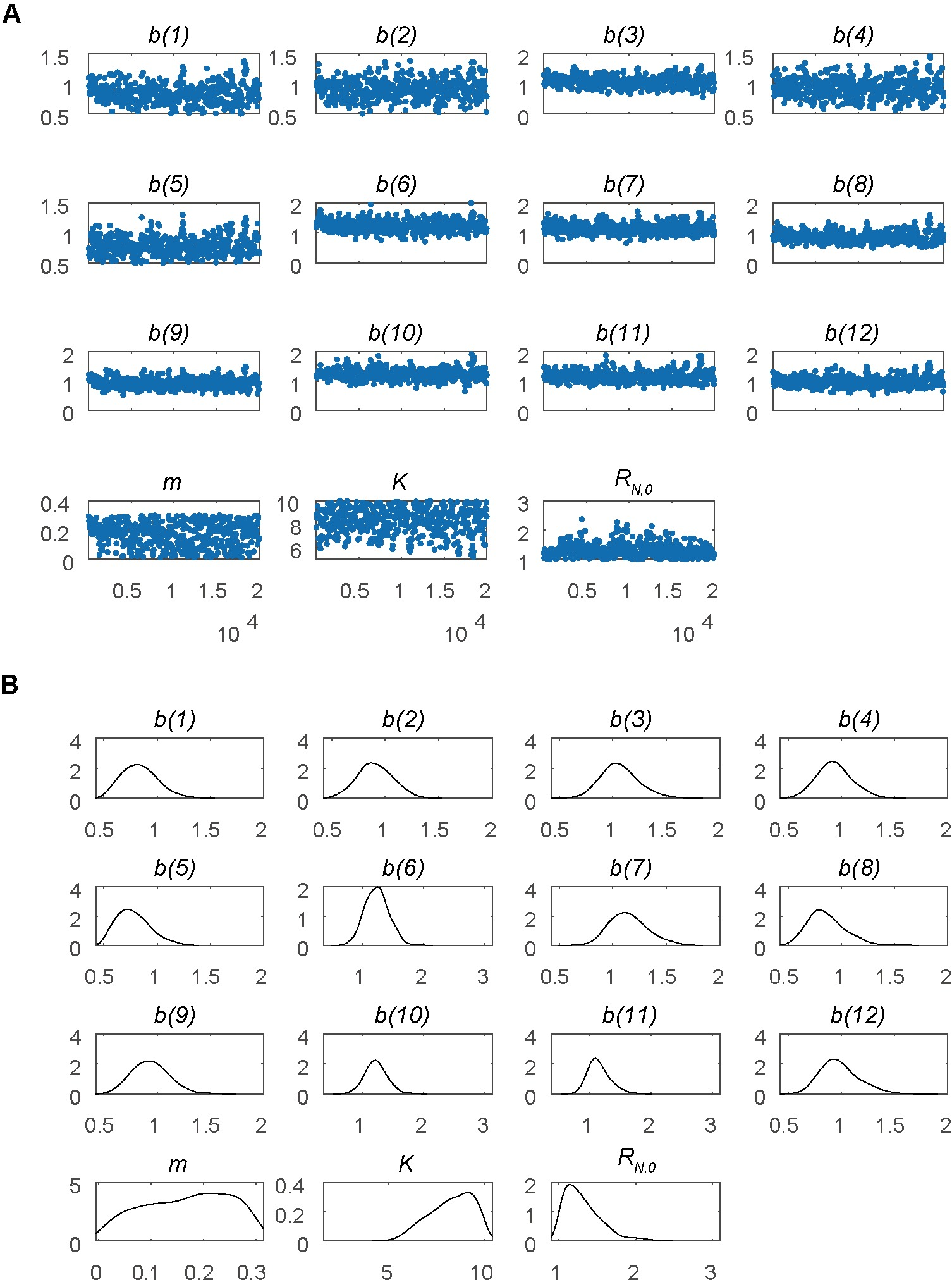

Supplement: S8 Fig — (A) The trace plot of markov chains of the parameters after 1 million burn-in. (B) The posterior distribution of the parameters after 1 million burn-in. All the estimations have passed the Gelman-Rubin-Brooks MCMC convergence diagnostic. (TIF) [file pntd.0007757.s008.tif]

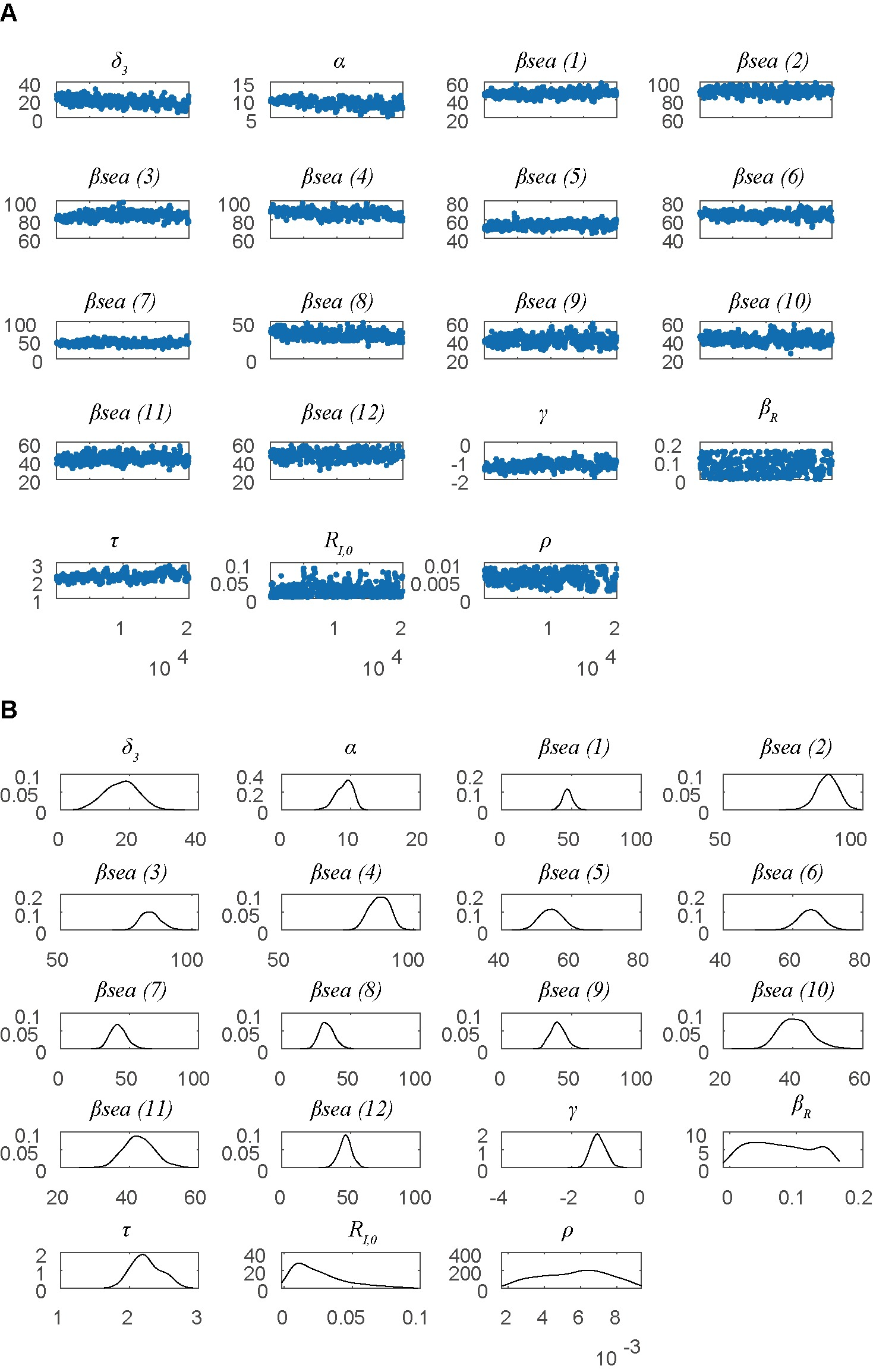

Supplement: S9 Fig — (A) The trace plot of markov chains of the parameters after 1 million burn-in. (B) The posterior distribution of the parameters after 1 million burn-in. All the estimations have passed the Gelman-Rubin-Brooks MCMC convergence diagnostic. (TIF) [file pntd.0007757.s009.tif]
